# Development of an anti-CAR antibody response in SIV-infected rhesus macaques treated with CD4-MBL CAR/CXCR5 T cells

**DOI:** 10.3389/fimmu.2022.1032537

**Published:** 2022-12-13

**Authors:** Brianna C. Davey, Mary S. Pampusch, Emily K. Cartwright, Hadia M. Abdelaal, Eva G. Rakasz, Aaron Rendahl, Edward A. Berger, Pamela J. Skinner

**Affiliations:** ^1^ Department of Veterinary and Biomedical Sciences, University of Minnesota, St. Paul, MN, United States; ^2^ Wisconsin National Primate Research Center, University of Wisconsin, Madison, Madison, WI, United States; ^3^ Laboratory of Viral Diseases, National Institute of Allergy and Infectious Diseases, National Institutes of Health, Bethesda, MD, United States

**Keywords:** CAR T cell, anti-drug antibodies (ADA), SIV, HIV, rhesus macaque, immunotherapy

## Abstract

T cells expressing a simian immunodeficiency (SIV)-specific chimeric antigen receptor (CAR) and the follicular homing molecule, CXCR5, were infused into antiretroviral therapy (ART) suppressed, SIV-infected rhesus macaques to assess their ability to localize to the lymphoid follicle and control the virus upon ART interruption. While the cells showed evidence of functionality, they failed to persist in the animals beyond 28 days. Development of anti-CAR antibodies could be responsible for the lack of persistence. Potential antigenic sites on the anti-SIV CAR used in these studies included domains 1 and 2 of CD4, the carbohydrate recognition domain (CRD) of mannose-binding lectin (MBL), and an extracellular domain of the costimulatory molecule, CD28, along with short linker sequences. Using a flow cytometry based assay and target cells expressing the CAR/CXCR5 construct, we examined the serum of the CD4-MBL CAR/CXCR5-T cell treated animals to determine that the animals had developed an anti-CAR antibody response after infusion. Binding sites for the anti-CAR antibodies were identified by using alternative CARs transduced into target cells and by preincubation of the target cells with a CD4 blocking antibody. All of the treated animals developed antibodies in their serum that bound to CD4-MBL CAR/CXCR5 T cells and the majority were capable of inducing an ADCC response. The CD4 antibody-blocking assay suggests that the dominant immunogenic components of this CAR are the CD4 domains with a possible additional site of the CD28 domain with its linker. This study shows that an anti-drug antibody (ADA) response can occur even when using self-proteins, likely due to novel epitopes created by abridged self-proteins and/or the self-domain of the CAR connection to a small non-self linker. While in our study, there was no statistically significant correlation between the ADA response and the persistence of the CD4-MBL CAR/CXCR5-T cells in rhesus macaques, these findings suggest that the development of an ADA response could impact the long-term persistence of self-based CAR immunotherapies.

## Introduction

Chimeric antigen receptor (CAR)-T cells have been successfully used as a cure strategy for cancers, primarily as a treatment for B cell leukemias and lymphomas (1–4). CAR T cells also show promise in treatment of viral diseases such as HIV through the recognition of envelope proteins on the surface of HIV-infected cells (5–8). Our studies have utilized a bispecific CAR, which contains domains 1 and 2 (D1/D2) of CD4, targeting the CD4 binding site on the viral envelope glycoprotein, gp120, and the carbohydrate recognition domain (CRD) of mannose-binding lectin (MBL) which targets the carbohydrates on the SIV envelope glycoproteins (9). Addition of the CRD of MBL both enhances potency of the CD4 CAR in a viral suppression assay and provides steric hindrance to the CD4 of the CAR to prevent viral entry in CD8+ CAR T cells (9). Since cytotoxic CD8 T cells are largely restricted from entry into lymphoid B cell follicles (10–14), where viral replication is most concentrated during HIV and SIV infection (10, 11, 15–20), the CD4-MBL CAR construct was modified to add the rhesus sequence for the follicular homing receptor, CXCR5 (21). T cells transduced to express CXCR5 migrate toward the chemokine ligand, CXCL13, *in vitro*, and accumulate in follicles *in vivo* (21, 22).

Our previous work infusing CD4-MBL CAR/CXCR5-T cells into SIV-infected rhesus macaques showed that these cells proliferated, accumulated in B cell follicles, and were associated with decreased viral loads in a subset of animals (23). However, we found that the CAR cells did not persist long-term *in vivo*, which may limit the efficacy of this treatment. Some CAR T cell studies have reported persistence, and functional persistence of CAR T cells *in vivo* for more than 10 years in humans (24, 25) and 2 years in rhesus macaques (24–26). However, in general, persistence remains a challenge for CAR T cell therapy, especially as a treatment for HIV (27, 28).

A potential limitation of CAR T-cell therapies is the development of an anti-drug antibody (ADA) response in the treated subject. These antibodies could limit the persistence of the CAR T-cells by activating complement-mediated killing or by antibody-dependent cellular cytotoxicity (ADCC). Anti-CAR antibody detection following CAR T cell treatment directed against single chain variable fragment CAR constructs has been reported in both humans (29, 30) and rhesus macaques (31). Because our CAR was derived from rhesus protein sequences and the MBL fragment lacked the variable regions, the CAR was considered unlikely to elicit an immune response (9, 21). However, each self-domain of the CAR is abridged and connected by a small non-self linker that may be immunogenic and could potentially induce an ADA response due to novel antigenic sites generated.

In this study, we investigated whether an ADA response was produced in rhesus macaques treated with CD4-MBL CAR/CXCR5-T cells. We found that anti-CAR Immunoglobulin G (IgG) antibodies were produced in all of the animals treated with CD4-MBL CAR/CXCR5-T cells. Using target cells with CAR variants, the data suggests that the antibody response is largely directed to the CD4 D1/D2 domains of the CAR, and partially to the CD28 transmembrane (TM) region and its linker. The antibodies were functionally capable of eliciting an ADCC response; however, we found no statistically significant correlation between the level of antibodies detected and the persistence of the CAR T cells. These findings suggest that an ADA response can occur, even when using self-proteins, due to the creation of novel epitopes. An anti-CAR response can potentially impact the long-term persistence of infused CAR T cells.

## Methods

### Animal study design and blood collection

The animals presented in this study were, in part, from a pilot study previously published (23) and, in part, from an unpublished study. All animal studies performed were reviewed and approved by the University of Wisconsin-Madison College of Letters and Sciences and Vice Chancellor for Research and Graduate Education Centers Institutional Animal Care and Use Committee (IACUC protocol number G005529). The rhesus macaques from the previously published study were in treatment groups 1 and 2 (T1 and T2) which were infected intrarectally with SIVmac251 (n=10) and treated with ART 63 to 68 days post-infection. The remaining animals, treatment group 3 (T3), were infected intrarectally with SIVmac239 (n=6) and ART was initiated at 30 to 75 days post-infection. ART was discontinued on the day of infusion and all animals had undetectable viral loads when they were treated with CD4-MBL CAR/CXCR5-T cells or used as control animals as described previously (23). The number of CD4-MBL CAR/CXCR5-T cells infused into the rhesus macaques varied due to experimental determination of the optimal therapeutic dose and to the expansion of the cells for each animal. Blood samples were collected before infusion, immediately after infusion, and 2, 6, 10, 14, 28, and 56 days post-treatment (DPT) and at necropsy. In addition, some animals were maintained up to 10 months post-treatment and blood was collected biweekly until necropsy. Peripheral blood mononuclear cells (PBMCs) were isolated by density gradient centrifugation, cryopreserved, and stored in liquid nitrogen. Serum samples were stored at -80°C. Target PBMCs were provided by Nonhuman Primate Biological Materials Distribution Core (NHPBMD).

### Cell production

For the production of therapeutic CD4-MBL CAR/CXCR5-T cells to treat animals, rhesus PBMCs were collected prior to SIV infection for T2 and T3 animal groups and following SIV infection for T1 animals. PBMCs were transduced as previously described (23, 32). In brief, cryopreserved cells were thawed and stimulated with plate-bound anti-CD3 and soluble anti-CD28 for two days prior to retronectin-mediated transduction with the CD4-MBL CAR/CXCR5 gammaretroviral vector. The cells were placed in G-Rex 6 well plates (Wilson Wolf Corporation) and expanded for 4 days prior to infusion into rhesus macaques. Mock transduced cells were subjected to the same stimulation and expansion but were not exposed to gammaretroviral vectors. The rhesus CD4-MBL CAR/CXCR5 construct was described previously (21, 23). For these studies, the same protocols were used with additional CAR constructs in order to produce gammaretroviral vectors and transduced PBMCs. The rhesus CD4-MBL 41BB CAR/CXCR5 construct was produced by adapting the rhesus CD4-MBL CD28 CAR/CXCR5 construct using rhesus-specific CD8 and 41BB sequences based on a patent for Chimeric Antigen-Modified T-Cells to Treat Cancer (33). The rhesus CARΔMBL is a rhesus adaptation of a CAR described in Ghanem et al., 2018 (9). Rhesus CXCR5 was produced using the reported sequence for Macaca mulatta transcript variant 1 (GenBank accession #XM_001100017).

### Flow cytometry

Multiparametric flow cytometry was carried out on thawed transduced, mock transduced or uncultured PBMCs using fluorescent tagged anti-human monoclonal antibodies that are cross-reactive to rhesus macaque proteins. Live/dead NIR (Invitrogen) was used to determine the viable cell population. The following antibodies were used in these studies: Alexa Fluor 700 mouse anti-human CD3 (SP34-2), Brilliant Violet 650 mouse anti-human CD4 (M-T477), PE mouse anti-human IgG (G18-145) (BD Biosciences), anti-human MBL (3E7) (Invitrogen) conjugated to Alexa Fluor 647 (Invitrogen), PE/Cy7 mouse anti-human CD20 (2H7) (BD Pharmingen), Brilliant Violet 785 mouse anti-human CD107a (H4A3) (Biolegend), and APC mouse anti-human CD159a (Z199) (Beckman Coulter). Samples were captured on CytoFlex (Beckman Coulter) and analyzed *via* FlowJo v10 (BD Life Sciences).

### Serum binding assay

The method used to detect anti-CAR antibodies was a modification of the procedure outlined by Potthoff et al. (34). Cryopreserved transduced CAR-T cells, as well as mock transduced PBMC, were thawed and used directly in all assays as target cells. Serum was heat inactivated for 30-35 minutes at 56°C, diluted fivefold in PBS, and added to wells containing 1x105 CAR or mock transduced T cells for a final dilution of 1:10. The serum and cells were incubated at 4°C for 20 minutes to allow binding of anti-CAR antibodies. The cells were washed twice to remove unbound antibodies of the serum. To measure bound IgG, cells were stained with live/dead NIR, anti-human CD3, anti-human CD4, anti-human MBL, anti-human CD20, and anti-human IgG. Following the 20-minute antibody stain, cells were fixed with 1% paraformaldehyde and captured by flow cytometry.

### CD4 blocking assay

CD4-MBL CAR/CXCR5 (CD28 TM CAR) transduced target cells were preincubated with BV650 anti-human CD4 for 30 minutes at room temperature and washed with PBS prior to incubation with the heat-inactivated serum and the detection procedure outlined above. Samples with and without anti-CD4 blocking were compared in the same assay.

### *In situ* hybridization and analysis

*In situ* hybridization was performed using RNAScope (Advanced Cell Diagnostics). The RNAScope protocol and analysis were described previously (23). Persistence and number of CD4-MBL CAR/CXCR5 cells in the follicle at 6 DPT were measured by RNAScope. Although the RNAScope data was reported previously (23), in this manuscript, the data was used in addition to new data from T3 animals to investigate possible correlations with the level of anti-CAR antibodies.

### Antibody-dependent cellular cytotoxicity assay

CD4-MBL CAR/CXCR5 transduced cells or mock transduced cells were used as target (T) cells and uninfected, uncultured rhesus PBMCs were used as effector (E) cells with an E: T ratio of 5:2. Serum was heat inactivated in the same manner as above, but was diluted for a final dilution of 1:115. Serum was added to thawed targets cells and incubated for 20 minutes to allow the anti-CAR antibodies to bind to the CAR cells, before adding uninfected PBMCs. Cells were treated with Brefeldin A (Biolegend) and mouse anti-human CD107a and incubated for 5 hours at 37°C. To measure CD107a degranulation by NK cells in the added PBMCs, cells were stained with live/dead NIR, anti-human CD3, anti-human CD20, anti-human IgG, and anti-human CD159a for 20 minutes. Cells were fixed with 1% paraformaldehyde and captured by flow cytometry.

### Statistical analysis

Association between continuous variables was assessed with Spearman’s correlation, using R version 4.2.0 (35).

## Results

### Anti-CAR IgG antibodies detected in the serum of CD4-MBL CAR/CXCR5 treated animals

**Figure 1:**
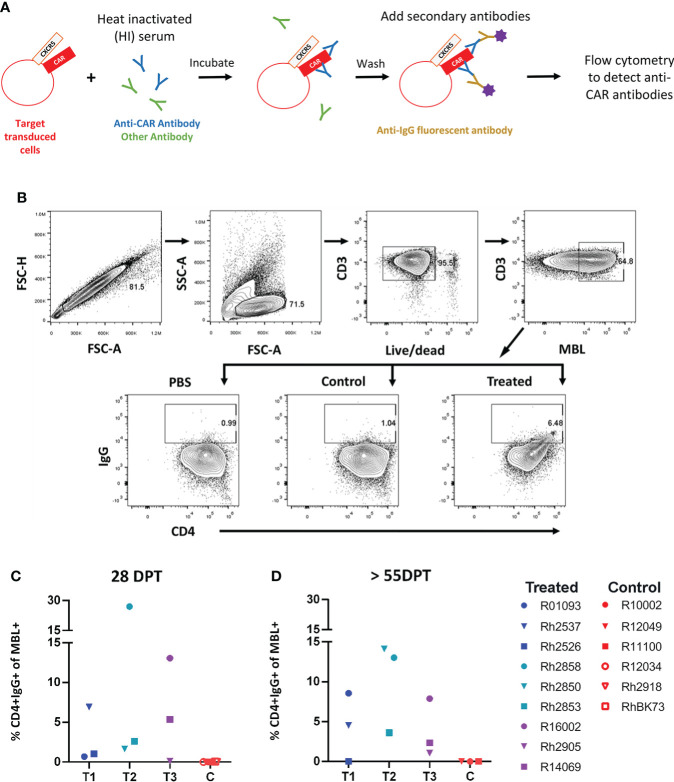
Anti-CAR antibodies detected at varying levels in the serum of all animals that received an infusion of CD4-MBL CAR/CXCR5 T cells. **(A)** Schematic showing antibody binding assay to determine levels of anti-CAR antibodies (percent CD4+IgG+). **(B)** Gating strategy to determine the amount of IgG bound to CD4-MBL CAR/CXCR5 transduced cells. Plots show singlets, lymphocytes, live CD3+, and MBL+. Within the MBL+ population, cells were analyzed for expression of CD4 and IgG. The representative treated animal is R14069 and the control animal is Rh2918 with serum collected at 28 days post-treatment. Data is reported after subtracting the level of IgG+ in PBS from serum samples (either treated or control). **(C)** The percent of CD4+IgG+ in treated animal serum (left) and control animal (right) at 4 weeks post-treatment and **(D)** 8 weeks post-treatment or necropsy (NX). NX ranges from 55 to 83 days post-treatment. Data is presented for T1 (blue), T2 (teal), T3 (purple) and control (red) animals.

We first examined serum from treated animals to determine whether they developed an anti-CAR antibody response to the infused CD4-MBL CAR/CXCR5 cells. To detect the presence of anti-CAR antibodies, we analyzed serum from treated animals using a modification of an *in vitro* flow cytometry assay previously described by Potthoff et al. and illustrated in **Figure 1A** (34). We determined the percentage of target CAR T cells that were bound with serum IgG after incubation with heat-inactivated serum from treated and control animals. Cells with bound IgG were detected by flow cytometry using the gating strategy outlined in **Figure 1B**. With the treated animal serum, we found varying levels of IgG bound to CD4+ MBL+ CAR-transduced cells, ranging from 0.5%-27% at an early time point, 28 DPT (**Figure 1C**), and 0-14% at later time points, ranging from 55 to 83 DPT (**Figure 1D**). In the control animals, we found no IgG bound to CD4+MBL+ CAR-transduced cells at either early or later time points (**Figures 1C, D**). Additionally, to ensure the animals did not have pre-existing antibodies to the CD4-MBL CAR/CXCR5 expressing cells, we evaluated the serum of the treated group pre-infusion with and we detected no bound IgG (**Supplementary Figure 1**).

### The CD4 D1/D2 domains appear to dominate the anti-CAR IgG response

**Figure 2:**
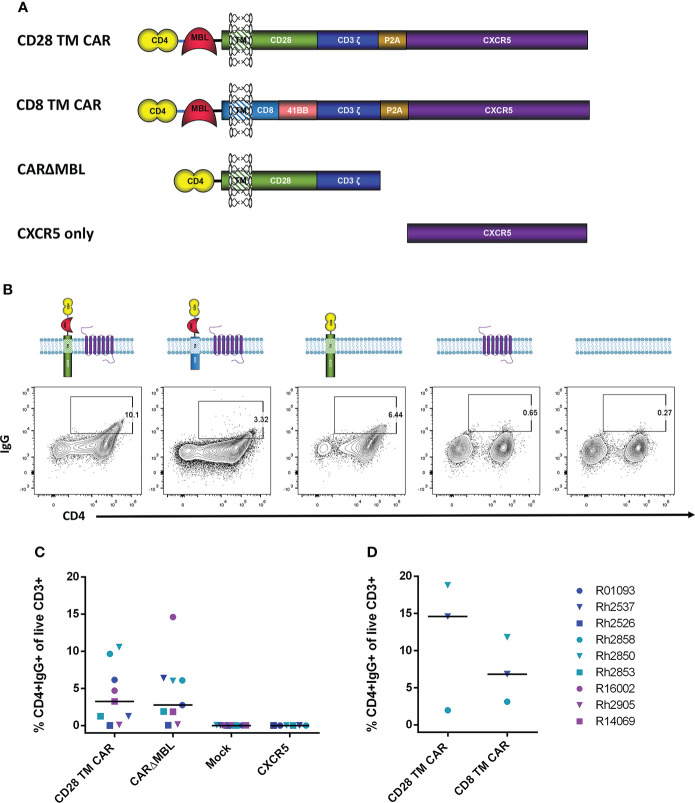
Serum IgG antibodies from treated animals bind CAR-expressing cells. **(A)** Schematic drawings of each construct. The original CD4-MBL CAR/CXCR5 (labeled CD28 TM CAR), a CD4-MBL CAR with a CD8 transmembrane domain (CD8 TM CAR), A CAR with CD4 D1/D2 and CD28 transmembrane domain (CARΔMBL) and CXCR5 only. P2A functions as a self-cleaving peptide to cleave the nascent CAR and CXCR5 polyprotein. CXCR5 encodes the endogenous rhesus CXCR5 protein. **(B)** Serum from animals was incubated with cells transduced with the indicated constructs and their expression at the cell surface. Representative flow cytometry plots showing the percent of CD4+IgG+ cells. Cells were gated on live CD3+ lymphocytes. **(C)** The percent of CD4+IgG+ cells in cells transduced with CD28 TM CAR (n=9), CARΔMBL (n=9), mock (n = 9), and CXCR5 only (n = 6) in treated animal serum from 28 to 83 DPT. Bars are drawn at the median. **(D)** The percent of CD4+IgG+ cells of cells transduced with the CD28 TM CAR (n = 3) and CD8 TM CAR (n=3) in treated animal serum from 70 to 168 DPT. Data is presented for T1 (blue), T2 (teal), T3 (purple) and control (red) animals.

The CD4-MBL CAR used in our animal studies contains the D1 and D2 domains of CD4 connected *via* a GGGGS linker to the CRD of MBL (9, 21, 23). The MBL is linked to the extracellular portion of the transmembrane (TM) protein CD28 *via* an AAA linker. In addition, for CD4-MBL CAR/CXCR5 T cells, a P2A cleavage site connects the CAR to the follicular homing receptor, CXCR5. This construct is shown in **Figure 2A**. To determine whether CXCR5 or one of the CD4-MBL CAR components might be immunogenic, we utilized target cells transduced with different CAR constructs or CXCR5 as shown in **Figure 2A**. For clarity and conciseness, the four different constructs will be designated according to the following: the CD28-based CAR will be referred to as the CD28 TM CAR, the CD8/41BB CAR will be referred to as the CD8 TM CAR, the CD28-based CAR without MBL will be referred to as CARΔMBL, and the CXCR5 only construct will be referred to as CXCR5 (**Figure 2A**). The amino acid sequences for each construct are presented in **Supplementary Figure 2**. Representative flow plots from serum IgG binding assays are shown in **Figure 2B**. Mock transduced target cells showed no serum IgG binding to target cells indicating serum IgG binding was specific to the CD4-MBL CAR/CXCR5 target cells (**Figure 2C**). To assess an IgG immune response to CXCR5, we used target cells expressing only CXCR5 and no CAR. We found no binding of the serum antibodies to the target cells expressing only CXCR5 (**Figure 2C**). The complete lack of binding to target cells transduced with only CXCR5 also suggests that gammaretroviral transduction alone did not lead to production of antibodies. In order to determine whether the MBL might contribute to IgG antibody binding, we compared the level of serum IgG binding to target cells expressing the CD28 TM CAR or the CARΔMBL, differing only in the removal of the MBL and the GGGGS linker. On average, we found similar levels of binding of the serum IgG antibodies to target cells expressing either the CD28 TM CAR or the CARΔMBL (**Figure 2C**), suggesting a lack of MBL antigenicity. Finally, to assess whether the serum IgG binding was specific for the extracellular portion of the CD28 molecule, which is connected to the MBL *via* a AAA linker, we utilized a CAR with a CD8 extracellular domain instead of CD28 in the serum binding assay. With serum from 2 of 3 animals, we found a decreased level of serum IgG bound to target cells without the CD28 extracellular portion of the CAR (**Figure 2D**), suggesting that the CD28 and its linker may contribute to the anti-IgG response detected in some animals.

**Figure 3:**
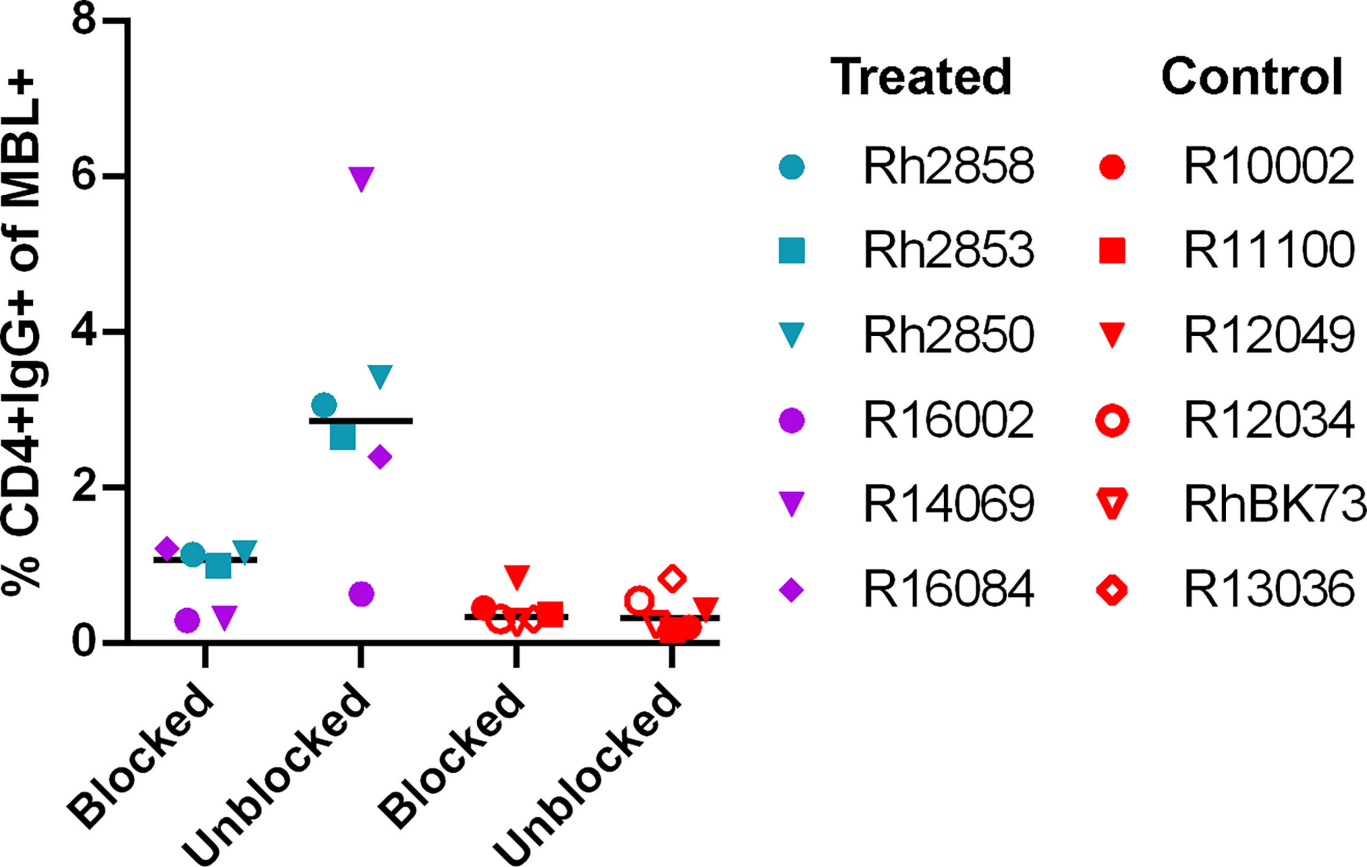
Anti-CD4 antibody blocks the majority of binding of anti-CAR antibodies to CAR T cells. The percent of CD4+IgG+ cells in treated animal serum (purple and teal symbols) or control (red symbols). CD4-MBL CAR/CXCR5 target cells were incubated with anti-CD4-BV650 antibody prior to incubation with heat-inactivated serum (blocked) or without prior incubation of anti-CD4-BV650 antibody (unblocked). Bars are drawn at the median. Data is presented for T1 (blue), T2 (teal), T3 (purple) and control (red) animals.

The potential immunogenicity of the CD4 D1/D2 domains was further evaluated by incubating the target cells with a labeled anti-CD4 antibody to block the CD4 domains before incubation with heat-inactivated serum. In the serum of treated animals, the level of IgG on target cells was reduced or eliminated in all six animals when the sites were blocked with anti-CD4 (**Figure 3**) suggesting that a major antigenic site in the CD4-MBL CAR is one or both of the CD4 domains or its linker to the MBL domain. The serum of control animals showed no change in binding with anti-CD4 treatment.

### Anti-CAR antibodies induce antibody-dependent cellular cytotoxicity against CD4-MBL CAR/CXCR5 cells *in vitro*

**Figure 4:**
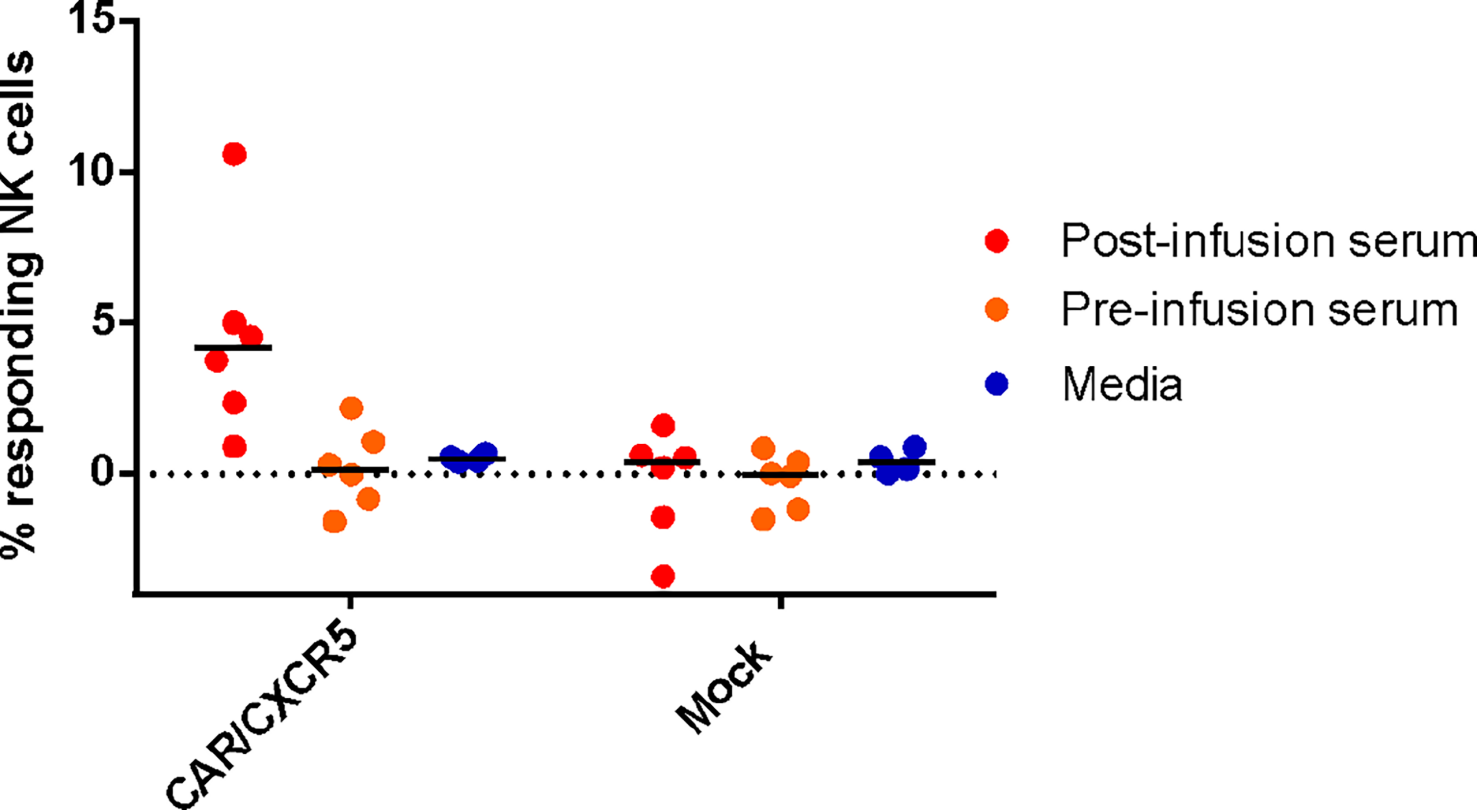
Anti-CAR antibodies bound to CD4-MBL CAR/CXCR5 cells induce antibody-dependent cellular cytotoxicity by NK cells. The percent of responding NK cells measured by the frequency of CD107a+ NK cells after co-culture with indicated serum (media n = 4, post-treatment n = 6, and pre-treatment serum n = 6) and target cell type (CD4-MBL CAR/CXCR5 or mock). Bars are drawn at the mean.

To assess the functionality of the antibodies against the CD4-MBL CAR/CXCR5 cells, serum was used in an antibody-dependent cellular cytotoxicity (ADCC) assay. Samples were analyzed for CD107a, a marker for functional degranulation, which correlates with target cell lysis by NK cells. We saw an increase in the degranulation of NK cells when serum from treated animals was incubated with CD4-MBL CAR/CXCR5 expressing target cells from 6 different animals (**Figure 4**). No measurable degranulation was detected with mock transduced cells, pre-infusion serum, or in samples that were tested without serum (media). These results suggest that the serum of treated animals contains functional antibodies that are capable of mediating ADCC.

### No statistically significant correlations between the level of anti-CAR antibodies and other factors

**Figure 5:**
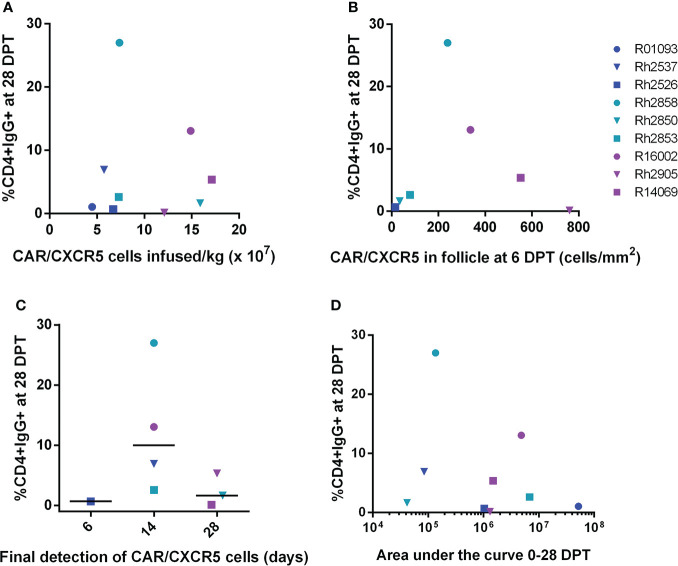
Anti-CAR antibody response at day 28 post-treatment versus the number of CD4-MBL CAR/CXCR5 cells infused/kg, peak level of CD4-MBL CAR/CXCR5 cells in B cell follicles, CD4-MBL CAR/CXCR5 persistence and viral load area under the curve. **(A)** Comparison of the level of IgG+CD4+ cells at 28 DPT and the number of MBL+CXCR5+ cells infused per kilogram (N = 9). **(B)** Comparison of the level of IgG+CD4+ cells at 28 DPT and number of CAR cells/mm^2^ in the follicle at peak (6DPT) (N = 7). No data is available for R01093 or Rh2537. **(C)** Relationship of IgG+CD4+ cells at 28 DPT and the last time point that CD4-MBL CAR/CXCR5 T cells were detected in the B cell follicles (N = 8). The persistence of CD4-MBL CAR/CXCR5 cells was defined as the last time point where CD4-MBL CAR/CXCR5 cells are detected in lymph node tissue at discrete biopsy time points. No data is available for R01093. Bars are drawn at the median. **(D)** Relationship of IgG+CD4+ cells at 28 DPT and the viral load area under the curve (AUC) from 0 to 28 DPT. All data were analyzed as a Spearman correlation. Data is presented for T1 (blue), T2 (teal), T3 (purple) and control (red) animals.

The levels of antibodies varied in the serum of the treated animals ranging from 0-27% (**Figures 1C** **, D**). Possible contributions to this variation are the number CAR/CXCR5 expressing cells infused per kilogram and the localization in B cell follicles. To explore the origin of this variation, we performed a series of correlation analyses between these factors and the level of anti-CAR antibodies in the serum. Treated animals were infused with differing numbers of CD4-MBL CAR/CXCR5 cells/kg ranging from 0.45 to 1.7 x 10^8^ CAR/CXCR5 T-cells/kg (**Supplementary Figure 3A**). The infused cells highly co-expressed MBL and CXCR5 (average, 67.0%; range, 55-79.4%; **Supplementary Figure 3B**) (23) and also had similar geometric mean fluorescence intensity (gMFI) of MBL staining on cells indicating similar levels of CARs on the surface of the cells (**Supplementary Figure 3C**). The cells also had a primary central memory phenotype (average, 64.4%; range 50.3-77.9; **Supplementary Figure 3D**) (23). We hypothesized that there may be an association between the number of CAR expressing cells infused and level of antibodies produced. However, when the level of CD4+ cells binding the serum IgG of treated animals at 28 DPT was analyzed as a function of infused CAR/CXCR5+ cells/kg (**Figure 5A**), our confidence interval for Spearman’s correlation was very wide, with an estimate of 0.18 (95% CI -0.55 to 0.76; p=0.65). Similarly, we found no statistically significant correlation between the level of CD4+ cells binding the serum IgG detectable at 56 DPT and dose of CAR/CXCR5+ cells (**Supplementary Figure 4A**). Spearman’s correlation was 0.07 (95% CI -0.63 to 0.7; p=0.88). There was also no significant correlation between the level of CAR on the surface of the infused cells (as measured by MBL gMFI) and the level of CD4+ cells binding serum IgG at 28DPT. The Spearman’s correlation was -0.33 (95% CI -0.82 to 0.44; p=0.39). Similarly, we found no statistically significant correlation between the level of CD4+ cells binding the serum IgG detectable at 56 DPT and MBL gMFI of the infusion cells. Spearman’s correlation was 0.00 (95% CI -0.66 to 0.66; p=1.00).

Since the amount of antigen presented to antibody-producing cells could vary depending upon the level of CAR T cells in the follicle, we evaluated whether there was a correlation between the peak number of CAR/CXCR5 expressing cells in the B cell follicle in the lymph node and the level of cells bound with anti-CAR IgG antibodies. As previously reported, 6 DPT was the peak of follicular localization of CD4-MBL CAR/CXCR5 T cells (23). When we evaluated the level of target cells bound with serum anti-CAR antibodies at 28 DPT relative to the peak number of CAR/CXCR5+ cells in the follicle, no statistically significant correlation was found (**Figure 5B**). Spearman’s correlation was 0.11 (95% CI -0.70 to 0.80; p=0.84). Likewise, no statistically significant correlation was found between the level of antibodies bound to target cells at 56 DPT and the peak number of CAR/CXCR5+ cells in the follicle (**Supplementary Figure 4B**). Spearman’s correlation was -0.14 (95% CI -0.81 to 0.68; p=0.78).

The presence of anti-CAR antibodies could potentially contribute to a lack of persistence of the CAR T cells so we examined whether there is a correlation between the level of antibodies in the serum and the persistence of the CD4-MBL CAR/CXCR5 cells in the lymph node B cell follicle. However, when the level of IgG bound to target cells at 28 DPT was analyzed as a function of the persistence of CAR cells (**Figure 5C**), the Spearman’s correlation was large, with an approximation of -0.25 (95% CI -0.81 to 0.55; p=0.55). We also noted no statistically significant correlation between persistence and the level of IgG bound to target cells at 56 DPT (**Supplementary Figure 3C**). Spearman’s correlation was 0.18 (95% CI -0.60 to 0.79; p=0.67).

To evaluate a potential association between overall viremia and production of an anti-CAR antibody response, we looked at the level of IgG bound to target cells as a function of the viral load area under the curve (AUC) from 0 to 28 DPT (**Figure 5D**) or 0 to 56 DPT (**Supplementary Figure 4D**). Again, the Spearman’s correlation was large with an approximation of -0.13 (95% CI -0.73 to 0.58, p=0.74) from day 0 to d28. We also noted no statistically significant Spearman’s correlation between the viral load AUC from d0 to d56 with an approximation of -0.27 (95% CI -0.80 to 0.49, p=0.49).

## Discussion

A challenge among many diseases and pathologies in the immunotherapy field has been to create effective, yet safe treatments. While therapeutic CAR T cells tend to be effective at killing SIV-infected cells *in vitro*, they often fail to persist *in vivo*, potentially due, in part, to the development of anti-CAR antibodies after infusion. Our CD4-MBL CAR/CXCR5 construct was designed with rhesus versions of self-proteins to minimize immunogenicity (21). In this study, we examined the serum of rhesus macaque post-CAR T cell infusion in order to assess the anti-drug antibody (ADA) response to the treatment.

The study provides evidence that the serum of rhesus macaques treated with CD4-MBL CAR/CXCR5 cells contains IgG antibodies that bind to target cells expressing the CAR and CXCR5 molecules. We focused on IgG since it was expected to be the primary immunoglobulin in serum at 28 DPT when we saw a complete loss of the CD4-MBL CAR/CXCR5-T cells in the blood and lymph nodes (23). The CD4-MBL CAR/CXCR5 transduced cells express a protein with D1/D2 domains of CD4, the CRD of MBL, and an extracellular domain of CD28, with linkers between the CD4 and MBL domains and between the MBL and CD28 domains, as well as the CXCR5 protein. Other than the linkers, all segments of the CAR are naturally occurring “self-proteins” in the rhesus macaque. However, a CAR, by nature, brings together multiple, novel antigenic sites. Linear epitopes can consist of as few as 6 to 9 amino acids but, in a majority of the cases, the surface amino acids in an antibody epitope are brought together by folding of the polypeptide chain which are discontinuous in the primary sequence and consist of an average of 14-19 amino acids (36–38).

While there could be a number of immunogenic sites in a polyclonal antibody response, we attempted to discern the primary immunogenic components of our CAR by utilizing alternative transduced targets. Overall, the antibodies bound equally to the cells containing a CAR with MBL and a CAR without MBL, though we saw increased binding with some serum samples and decreased binding with others. We conclude that the MBL carbohydrate recognition domain of the CAR does not appear to be a primary immunogenic site. While the extracellular portion of the CD28 molecule and its AAA linker may contain immunogenic sites and may be responsible for some of the antibody binding, it is unlikely that it is solely responsible for the immunogenicity of the CAR since detectable antibody binding was still observed when a CAR lacking the CD28 domain was used as a target.

By utilizing CXCR5 only transduced T cells, and detecting no immunoglobulin binding, we were able to rule out CXCR5 as a potential immunogen for the ADA response. The lack of immunogenicity was expected since the CXCR5 was of rhesus origin and was not part of a fusion protein. Additionally, the CXCR5 only transduced cells allow us to address the possibility that cells that are genetically modified by viral transduction may retain some residual viral proteins on their surface which may prove to be antigenic (28). The lack of antibody binding suggests that the transduction procedure itself did not generate antigenic sites on the surface of the infusion cell product. However, we cannot rule out the possibility that epitopes from the gammaretroviral transduction were able to elicit a cell-mediated immune response leading to a destruction of the CAR T cells *in vivo*, as seen with anti-carbonic anhydrase IX-CAR T cell therapy (30).

Although the CAR utilizes self-domains of CD4 (9, 21, 39), we assessed the potential antigenicity by blocking the CD4 sites on the target cells prior to incubation with treated animal serum. We found a reduction or loss of binding with CD4 blocking in all animals indicating that the majority of the antibodies were binding to the CD4 domains of the CAR. Since the CD4 antibody may also sterically hinder adjacent regions, we cannot rule out a blockage of an adjacent epitope, especially the CD4-linker junction. However, we have found that we can detect the binding of MBL antibodies to the CD4-MBL CAR in anti-CD4 blocked cells by flow cytometry (21, 23) so it is unlikely that the CD4 antibody is blocking the MBL or CD28 regions of the CAR. The finding that blocking D1D2 on the CAR with anti-CD4 antibody (M-T477) reduced the detection of ADA responding antibodies is intriguing since the D1/D2 domains of CD4 also function as the HIV/SIV recognition site on CD4. Visualization studies have demonstrated that pretreatment with M-T477 reduces the number of virions binding to the surface of T cells exposed to HIV in a dendritic cell-T cell synapse (40). Since the ADA response is targeting the SIV binding site on CD4, it could potentially block the binding of the CD4 CAR T cells to HIV or SIV-infected cells thus reducing their efficacy and expansion as was seen in a study with anti-SIV Env CAR T cells (31). Evaluation of serum of treated animals for an ADA response is an essential step in CAR T cell pre-clinical trials. Several reported HIV CAR T cells are designed with CD4 domains (5, 6, 8, 41) and their infusion may also lead to ADA responses, potentially limiting the effectiveness of the anti-HIV CAR T cells.

Our previous work has shown that our CD4-MBL CAR/CXCR5 T cells migrate from the circulating blood into the lymph nodes less than 1 week following infusion (23). The transduced cells traffic into the B cell follicle where large numbers of secondary B cells reside (42), increasing the chance of antigen recognition and initiation of an antibody response. However, we found no indication that either the number of CAR T cells infused, or the number of CAR T cells in the follicle at 6 days, led to increased antibody production. Of note, the large confidence intervals in these correlations indicate that we cannot conclude any association, either positive or negative. It is possible that, with additional animal subjects, we could more conclusively determine a possible impact of cell number or localization on antibody production.

The anti-CAR antibodies demonstrated functional killing in the *in vitro* ADCC assay, which suggests a possible mechanism of *in vivo* clearance. If the antibody was functioning in the removal of the anti-SIV CAR T cells, we might expect to see an association between the level of the anti-CAR antibody in the serum and level of viremia in the treatment animals. However, we were unable to find a correlation between the amount of antibody and the viral load in the treatment animals. Additionally, we were unable to conclude any association between the persistence of CAR T cells in the treated animals and the level of antibody in the serum. We did note that animal Rh2526 poses as an outlier, persisting only until day 6. If that animal is excluded, we observe a negative association between the level of anti-CAR antibodies and persistence of the CAR cells, with lower antibody levels at 28 days post-treatment associated with longer CAR-T cell persistence. This association suggests that these anti-CAR antibodies may play a role in the *in vivo* clearance of the CD4-MBL CAR/CXCR5 T cells. However, examination of additional treated animals may be necessary to comprehensively assess the effect of ADA on *in vivo* persistence. Conversely, two CAR T cell cancer medications, which are currently in clinical use, produce an ADA response without a negative impact. Research with Yescarta and Kymriah, products currently approved for use in patients with large B cell lymphoma and acute lymphoblastic anemia respectively, has demonstrated a lack of correlation between anti-CAR antibody level and expansion and persistence of the CAR T cells (28, 43–45). Nevertheless, the impact of ADA responses can be variable, and therefore determining the relative immunogenicity in preclinical models is paramount to developing a safe and effective therapy (46).

In the case of our CD4-MBL CAR/CXCR5 T cells, the lack of persistence of the cells in the study animals may be a complex interaction between the ADA in the serum and, possible infection of the CAR T cells by SIV, exhaustion of the CAR T cells post-treatment, the low level of antigen present at the time of ART release, or potential cell-mediated CAR-T cell clearance. It is somewhat unlikely that the infection of the CAR T cells by SIV and subsequent cell death is a major contributor to low persistence of our CD4-MBL CAR/CXCR5 T cells. The CD4 domains in our CAR do not act as an entry receptor due to the MBL CRD which blocks entry of HIV or SIV due to steric hindrance (9). Thus, while naturally occurring CD4+ CD4-MBL CAR-T cells could be infected and eliminated, the CD8+ CD4-MBL CAR-T cells are protected from infection (9). Additionally, our previous studies have shown that we rarely detect CAR SIV-infected cells by RNAscope (23). While it is possible that the CD4-MBL CAR/CXCR5 T cells have become exhausted *in vivo*, they have a high central memory population at the time of infusion, a phenotype known to be associated with better persistence (47). Further analysis of the CD4-MBL CAR/CXCR5 T cells from post-treatment biopsies may allow a better determination of the cell phenotype over time. The low level of the vial envelope antigen at the time of ART interruption poses as a barrier to antigen stimulation of HIV/SIV CAR T cell treatments (23, 48, 49). Other studies have reported that infusion of SIV Env expressing cells after CAR T cell infusion promoted *in vivo* expansion of CAR T cells (49). Future studies including SIV Env infusion at the time of CAR T cell infusion could elucidate if the mechanism is due to anti-CAR antibody-mediated clearance or lack of sufficient antigen stimulation. Finally, it is possible that cell-mediated clearance or the complement pathway led to CAR T cell elimination. Further studies of the early adaptive immune response that reach further than this study of the humoral response could shed more light on the mechanisms of clearance.

This study indicates that, when developing chimeric antigen receptors, investigators should be aware that the secondary and tertiary structures of a CAR may allow the development of novel antigenic sites even if using self-proteins. Unavoidable junctions for assembly of these proteins can act as foreign antigens and increase the likelihood of immunogenicity. ADA responses may impact the persistence of CAR-T cells and thus efficacy. Efforts to increase persistence by improving memory phenotypes of the cell product, reducing exhaustion, or increasing antigenic stimulation in low antigenic settings may be met with low *in vivo* persistence if an ADA response is elicited. The development of anti-CAR antibodies could require the use of a different CAR with different extracellular domains for retreatment of a patient with CAR-T cell therapy.

## Data availability statement

The datasets presented in this study can be found in online repositories. The names of the repository/repositories and accession number(s) can be found below: Data Repository for U of M (DRUM), https://hdl.handle.net/11299/243377.

## Ethics statement

The animal study was reviewed and approved by University of Wisconsin-Madison College of Letters and Sciences and Vice Chancellor for Research and Graduate Education Centers Institutional Animal Care and Use Committee (IACUC protocol number G005529).

## Author contributions

BD, MP, EC, EB and PS contributed to the conceptualization of the study. BD and EC contributed to the methodology. BD, MP, EC and AR contributed to formal analysis of the data. BD, MP, and HA contributed to investigation. BD was responsible for data curation. ER, EB and PS provided supervision for the study. ER and EB provided resources for the study. ER, EB and PS provided funding for the study. PS provided project administration for the study. BD, MP and PS were responsible for writing the manuscript. All authors contributed to manuscript revision, read and approved the submission.

## Supplementary material

Supplementary Figure 1 No anti-CAR antibody detected in the serum before infusion of CD4-MBL CAR/CXCR5 T cells. The level of CD4+IgG+ in treated animal serum (left) and control animal serum (right) at 0 DPT. Serum samples were collected 10 minutes before infusion of cells. Data is presented for T1 (blue), T2 (teal), T3 (purple) and control (red) animals.

Supplementary Figure 2 Amino acid sequences for the constructs used in these studies. **(A)** The rhesus CD4-MBL CD28 CXCR5 sequence was previously described (21). **(B)** The rhesus CD4-MBL 41BB CAR CXCR5 was produced by adapting the rhesus CD4-MBL CD28CAR CXCR5 using rhesus specific CD8 and 41BB sequences based on a patent for Chimeric Antigen-Modified T-Cells to Treat Cancer (33). **(C)** The Rhesus CARΔMBL is a rhesus adaptation of a CD4-MBL CAR previously described (9). **(D)** Rhesus CXCR5 was produced using reported sequence for Macaca mulatta transcript variant 1 (GenBank accession #XM_001100017).

Supplementary Figure 3 CD4-MBL CAR/CXCR5 T cells infused into rhesus macaques. T1 animals (blue), T2 animals (teal), and T3 animals (purple). **(A)** The number of MBL+CXCR5+ cells infused per kg body weight of the treated animal. **(B)** Co-expression of the CAR, measured by MBL+, and CXCR5 on the infusion cells. **(C)** Expression of the CAR, measured by gMFI of MBL on the infusion cells. **(D)** The central memory phenotype of the infused cells as measured by CD95-CD28+ after gating on live, CD3+, CD8+ populations.

Supplementary Figure 4 Anti-CAR antibody response at day 56 post-treatment versus the number of CD4-MBL CAR/CXCR5 cells infused/kg, peak level of CD4-MBL CAR/CXCR5 cells in B cell follicles, CD4-MBL CAR/CXCR5 persistence and viral load area under the curve. **(A)** Comparison of level of IgG+CD4+ cells at 56 DPT and the number of MBL+CXCR5+ cells infused per kilogram (N=9). **(B)** Comparison of the level of IgG+CD4+ cells at 56 DPT and the number of CAR cells/mm^2^ in the follicle at peak (6DPT) (N=7). No data is available for R01093 or Rh2537. **(C)** Relationship of IgG+CD4+ cells at 56 DPT and the last time point that CD4-MBL CAR/CXCR5 T cells were detected in the B cell follicles (N=8). The persistence of CD4-MBL CAR/CXCR5 cells was defined as the last time point where CD4-MBL CAR/CXCR5 cells are detected in lymph node tissue at discrete biopsy time points. No data is available for R01093. Bars are drawn at the median. **(D)** Relationship of IgG+CD4+ cells at 56 DPT and the viral load area under the curve (AUC) from 0 to 56 DPT. All data were analyzed as a Spearman correlation. Data is presented for T1 (blue), T2 (teal), T3 (purple) and control (red) animals.
