# Supplementary figures and images for: Development of an anti-CAR antibody response in SIV-infected rhesus macaques treated with CD4-MBL CAR/CXCR5 T cells
Source: Front Immunol. 2022 Dec 13;13:1032537. doi: 10.3389/fimmu.2022.1032537 (PMC9793449; doi:10.3389/fimmu.2022.1032537)

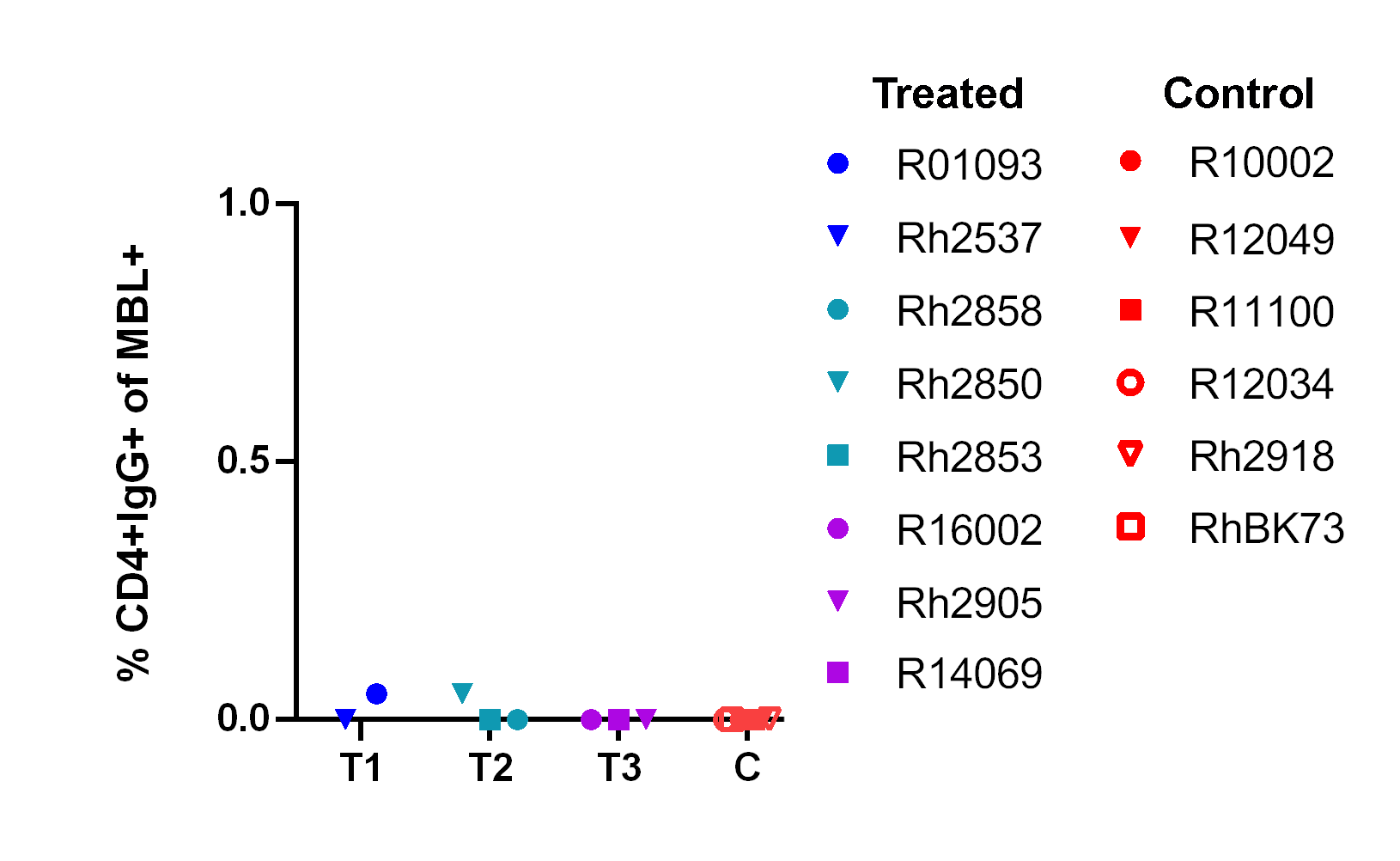

Supplement: Supplementary Figure 1 — No anti-CAR antibody detected in the serum before infusion of CD4-MBL CAR/CXCR5 T cells. The level of CD4+IgG+ in treated animal serum (left) and control animal serum (right) at 0 DPT. Serum samples were collected 10 minutes before infusion of cells. Data is presented for T1 (blue), T2 (teal), T3 (purple) and control (red) animals. [file Image_1.tif]

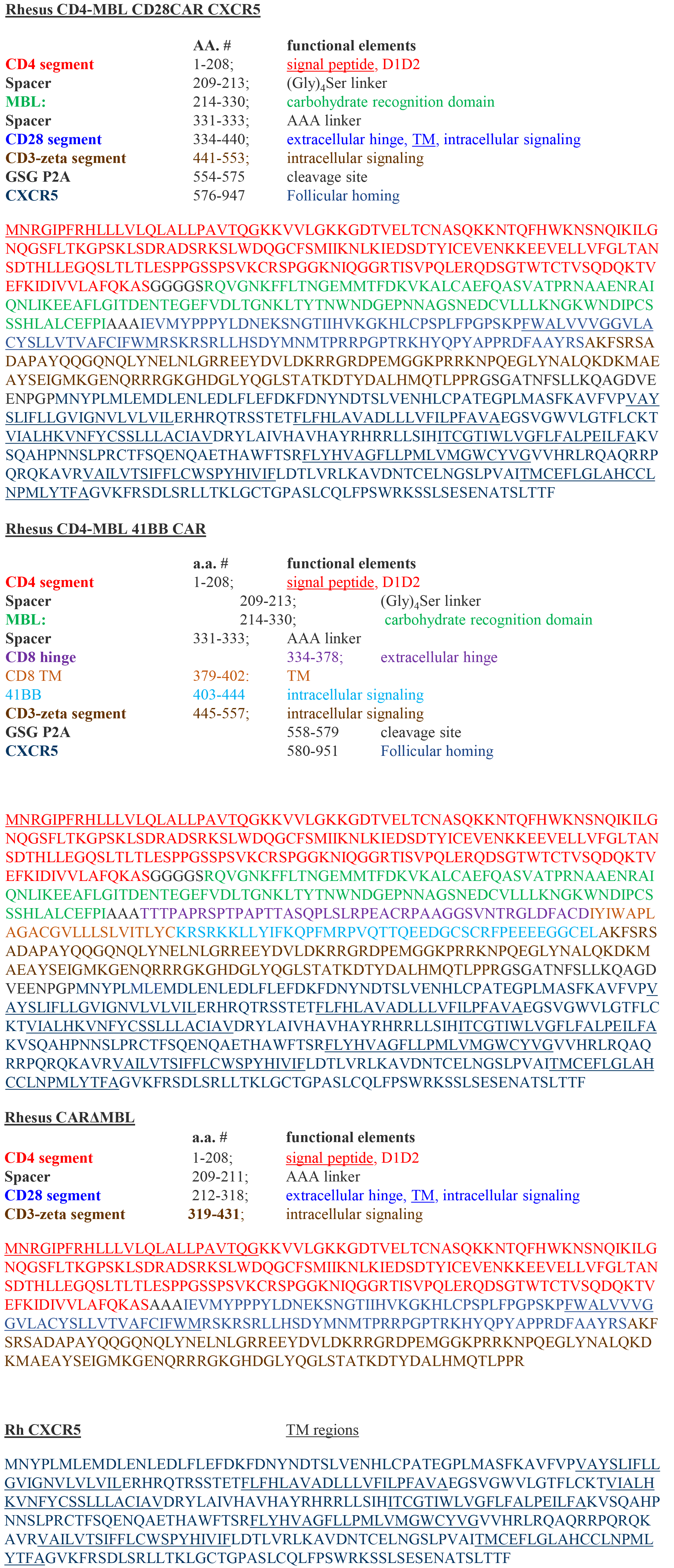

Supplement: Supplementary Figure 2 — Amino acid sequences for the constructs used in these studies. (A) The rhesus CD4-MBL CD28 CXCR5 sequence was previously described (21). (B) The rhesus CD4-MBL 41BB CAR CXCR5 was produced by adapting the rhesus CD4-MBL CD28CAR CXCR5 using rhesus specific CD8 and 41BB sequences based on a patent for Chimeric Antigen-Modified T-Cells to Treat Cancer (33). (C) The Rhesus CARΔMBL is a rhesus adaptation of a CD4-MBL CAR previously described (9). (D) Rhesus CXCR5 was produced using reported sequence for Macaca mulatta transcript variant 1 (GenBank accession #XM_001100017). [file Image_2.tif]

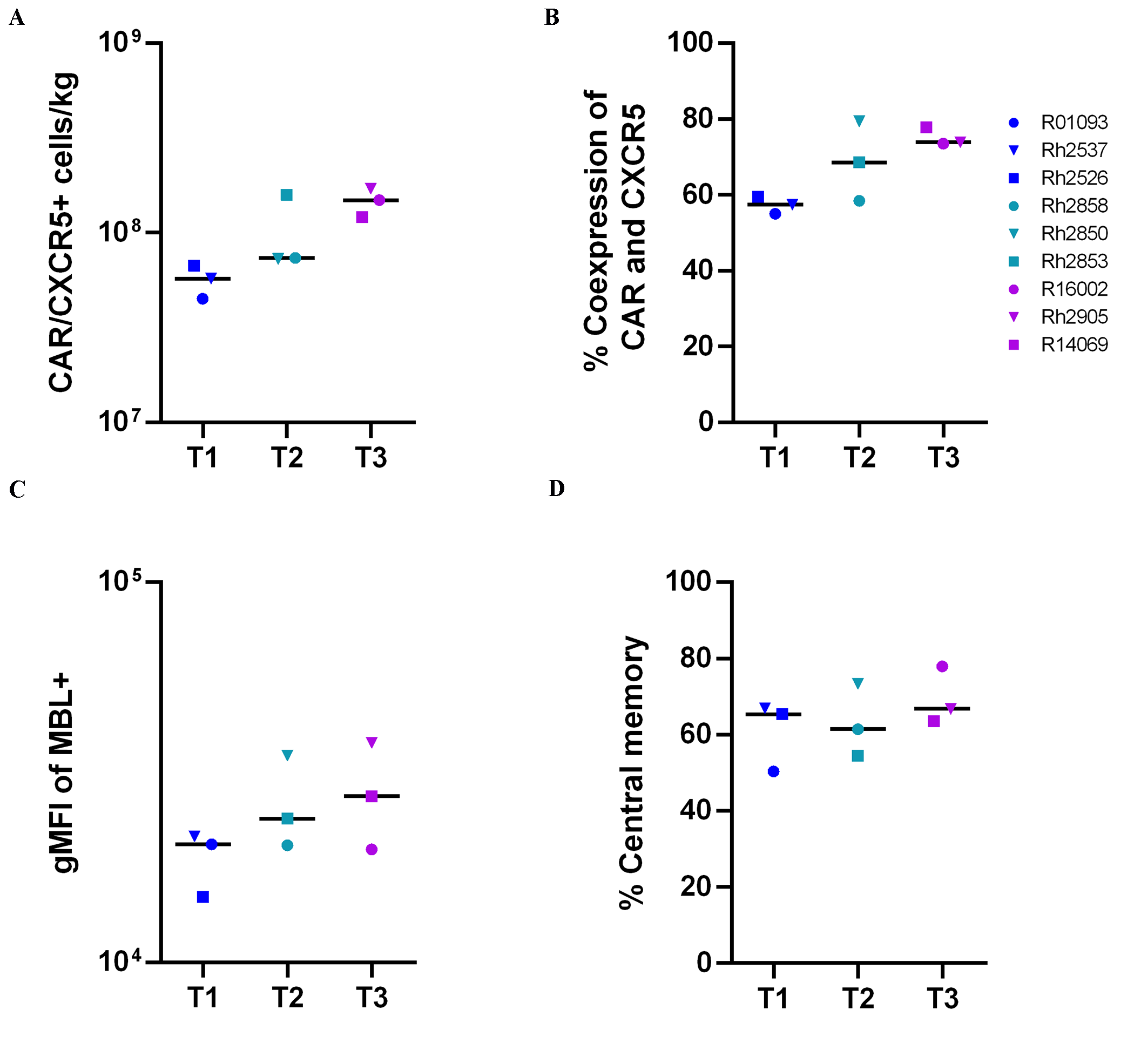

Supplement: Supplementary Figure 3 — CD4-MBL CAR/CXCR5 T cells infused into rhesus macaques. T1 animals (blue), T2 animals (teal), and T3 animals (purple). (A) The number of MBL+CXCR5+ cells infused per kg body weight of the treated animal. (B) Co-expression of the CAR, measured by MBL+, and CXCR5 on the infusion cells. (C) Expression of the CAR, measured by gMFI of MBL on the infusion cells. (D) The central memory phenotype of the infused cells as measured by CD95-CD28+ after gating on live, CD3+, CD8+ populations. [file Image_3.tif]

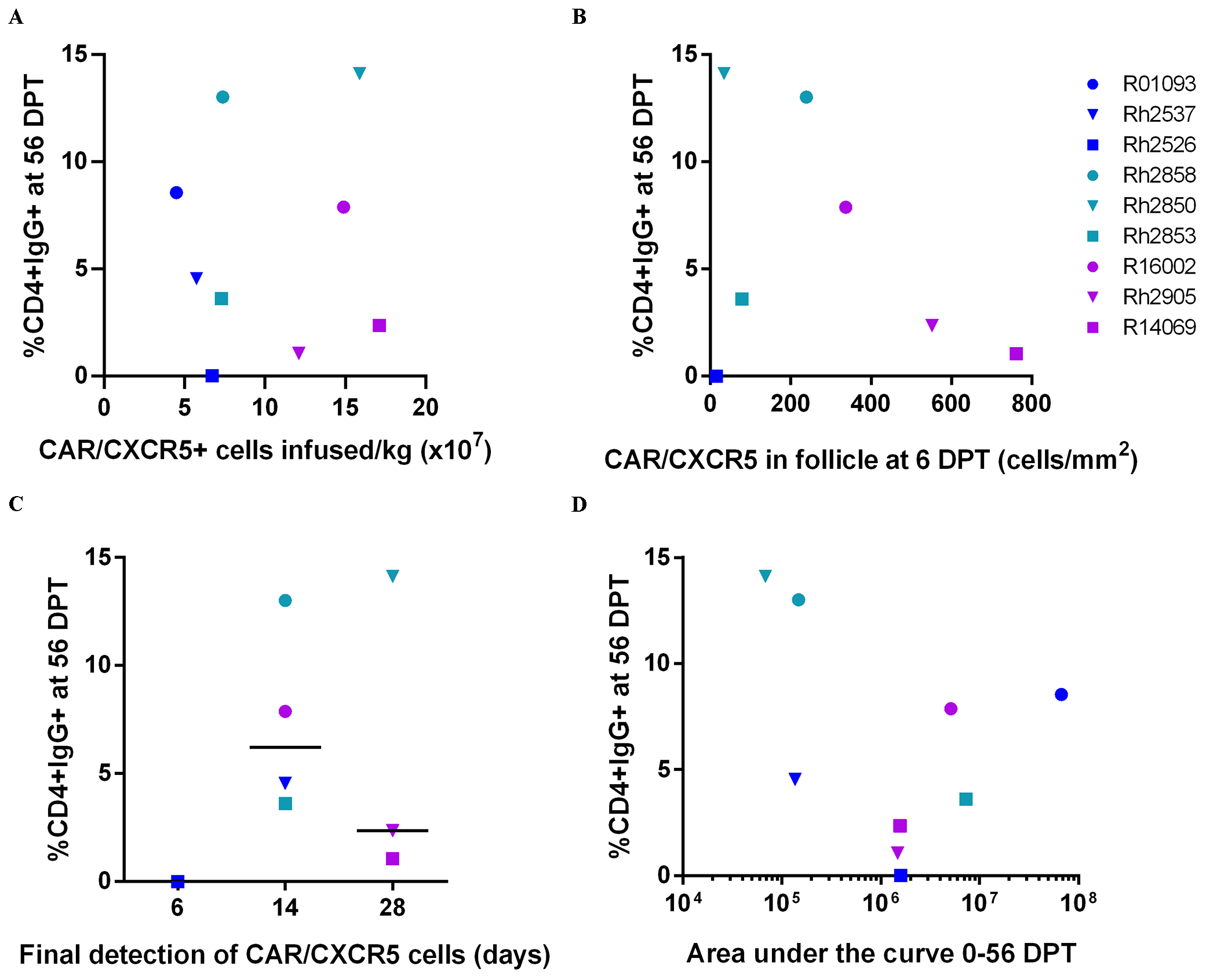

Supplement: Supplementary Figure 4 — Anti-CAR antibody response at day 56 post-treatment versus the number of CD4-MBL CAR/CXCR5 cells infused/kg, peak level of CD4-MBL CAR/CXCR5 cells in B cell follicles, CD4-MBL CAR/CXCR5 persistence and viral load area under the curve. (A) Comparison of level of IgG+CD4+ cells at 56 DPT and the number of MBL+CXCR5+ cells infused per kilogram (N=9). (B) Comparison of the level of IgG+CD4+ cells at 56 DPT and the number of CAR cells/mm2 in the follicle at peak (6DPT) (N=7). No data is available for R01093 or Rh2537. (C) Relationship of IgG+CD4+ cells at 56 DPT and the last time point that CD4-MBL CAR/CXCR5 T cells were detected in the B cell follicles (N=8). The persistence of CD4-MBL CAR/CXCR5 cells was defined as the last time point where CD4-MBL CAR/CXCR5 cells are detected in lymph node tissue at discrete biopsy time points. No data is available for R01093. Bars are drawn at the median. (D) Relationship of IgG+CD4+ cells at 56 DPT and the viral load area under the curve (AUC) from 0 to 56 DPT. All data were analyzed as a Spearman correlation. Data is presented for T1 (blue), T2 (teal), T3 (purple) and control (red) animals. [file Image_4.tif]
